# Use of biliary metal stents and defect-closure systems: a new strategy to treat refractory benign esophageal strictures

**DOI:** 10.1055/a-2729-2107

**Published:** 2025-11-14

**Authors:** Matteo Colombo, Antonio Capogreco, Valeria Poletti, Giacomo Marcozzi, Marco Spadaccini, Alessandro Fugazza, Alessandro Repici

**Affiliations:** 1 551905 Endoscopy Unit, IRCCS Humanitas Research Hospital, Rozzano, Milan, Italy; 2 437807 Department of Biomedical Sciences, Humanitas University, Pieve Emanuele, Milan, Italy

Benign esophageal strictures typically present with symptoms such as dysphagia, regurgitation, weight loss, and chills. They are most commonly caused by chronic acid reflux, caustic injuries, eosinophilic esophagitis, and iatrogenic trauma from endoscopic or surgical procedures 1 2 . First-line treatment involves endoscopic sessions of mechanical or pneumatic dilatation 3 4 .

In refractory cases, temporary placement of self-expandable metal stents (SEMSs) may be considered. Fully covered SEMSs are preferred due to their lower risk of embedment and ease of removal 5 . However, in cases of severe stenosis, conventional esophageal SEMSs may be too large for safe deployment. In such situations, SEMSs with smaller diameters may be used, though they carry a higher risk of migration. Stent fixation can mitigate this risk. Yet, dedicated fixation devices (e.g., Stent Fix, Ovesco) may be too invasive in delicate areas like the pharynx, and suture systems (e.g., OverStitch, Apollo) can be challenging to use in narrow anatomical regions. The X-tack closure device (Endoscopic HeliX Tacking System, Boston Scientific) may offer a less invasive, user-friendly alternative.

We report the case of a 49-year-old male with a severe pharyngeal stricture following total laryngectomy and partial pharyngectomy for squamous cell carcinoma ( Video 1 ). Despite multiple mechanical and pneumatic dilations, only partial improvement was achieved. A fully covered biliary SEMS (10 mm × 80 mm) was placed across the stenosis. Four HeliX tacks were applied for fixation: two through the stent mesh and two directly into the pharyngeal tissue, anchoring deeply in the submucosal and muscular layers. Final suture tension and secure locking were achieved using a suture cinch system.

Deployment of a fully-covered, 10 mm × 80 mm, biliary SEMS over the stenotic tract of a severe pharyngeal stricture. Subsequent stent fixation through X-tack device. SEMS, self-expandable metal stent.Video 1

This case represents the first successful use of a biliary SEMS with X-tack fixation to treat a severe, refractory benign esophageal stricture.

Endoscopy_UCTN_Code_TTT_1AO_2AH

## Notes

Endoscopy E-Videos https://eref.thieme.de/e-videos
*E-Videos* is an open access online section of the journal *Endoscopy* , reporting on interesting cases and new techniques in gastroenterological endoscopy. All papers include a high-quality video and are published with a Creative Commons CC-BY license. Endoscopy E-Videos qualify for HINARI discounts and waivers and eligibility is automatically checked during the submission process. We grant 100% waivers to articles whose corresponding authors are based in Group A countries and 50% waivers to those who are based in Group B countries as classified by Research4Life (see: https://www.research4life.org/access/eligibility/ ). This section has its own submission website at https://mc.manuscriptcentral.com/e-videos .
